# Ammonium Phosphate as Inhibitor to Mitigate the Corrosion of Steel Rebar in Chloride Contaminated Concrete Pore Solution

**DOI:** 10.3390/molecules25173785

**Published:** 2020-08-20

**Authors:** Soumen Mandal, Jitendra Kumar Singh, Dong-Eun Lee, Taejoon Park

**Affiliations:** 1 Intelligent Construction Automation Center, Kyungpook National University, 80, Daehak-ro, Buk-gu, Daegu 41566, Korea; sou.chm@gmail.com; 2 Innovative Durable Building and Infrastructure Research Center, Department of Architectural Engineering, Hanyang University, 1271 Sa3-dong, Sangrok-gu, Ansan 15588, Korea; jk200386@hanyang.ac.kr; 3 School of Architecture, Civil, Environment, and Energy, Kyungpook National University, 1370, Sangyegk-Dong, Buk-Gu, Daegu 702-701, Korea; 4 Department of Robotics Engineering, Hanyang University, 55 Hanyangdaehak-ro, Ansan, Gyeonggi-do 15588, Korea

**Keywords:** steel, corrosion, inhibitor, concrete, potentiodynamic polarization, electrochemical impedance spectroscopy, scanning electron microscopy, raman spectroscopy

## Abstract

In the present study, different amounts, i.e., 1–3 *v*/*v*% of 1 M ammonium phosphate monobasic, were used as an eco-friendly corrosion inhibitor to mitigate the corrosion of steel rebar exposed to simulated concrete pore (SCP) + 3.5 wt% NaCl solution at a prolonged duration. Potentiodynamic polarization results show that as the amount of inhibitor is increased, the corrosion resistance of steel rebar is increased. The steel rebar exposed to 3% inhibitor-containing SCP + 3.5 wt% NaCl solution exhibited nobler corrosion potential (*E_corr_*), the lowest corrosion current density (*i_corr_*), and 97.62% corrosion inhibition efficiency after 1 h of exposure. The steel rebars exposed to 3% inhibitor-containing SCP + 3.5 wt% NaCl solution revealed higher polarization resistance (*R_p_*) and film resistance (*R_o_*) with exposure periods compared to other samples owing to the formation of passive film. The scanning electron microscopy (SEM) of steel rebar exposed to 3% inhibitor-containing SCP + 3.5 wt% NaCl solution showed homogenous and uniform dendritic passive film which covers all over the surface, whereas, bare, i.e., SCP + 3.5 wt% NaCl solution exposed samples exhibited pitting and irregular morphology. Raman spectroscopy results confirm the formation of goethite (α-FeOOH), maghemite (γ-Fe_2_O_3_), and iron phosphate (FePO_4_) as a passive film onto the steel rebar surface exposed to 3% inhibitor-containing SCP + 3.5 wt% NaCl solution. These phases are responsible for the corrosion mitigation of steel rebar which are very protective, adherent, and sparingly soluble.

## 1. Introduction

The reinforced concrete is an important part of the fiscal development as it is widely used in building materials. However, corrosion causes the early age degradation of steel reinforced concrete and weakens the structure, which results in a very high global economic loss [1,2,3,4]. The steel rebar is a key constituent that provides structural durability to the concrete. Once the corrosion starts, the durability of the concrete reduces. The hydrolysis of Na, K, Ca, and Si oxides present in concrete makes the environment alkaline and favors the formation of passive film onto the steel surface [5,6]. The formation of passive film depends on temperature, humidity, composition of steel bar, composition of concrete, aggregates, etc. However, the localized corrosion of the reinforced steel occurs owing to the increase in chloride concentration and acidification of the concrete at the steel/concrete interface [1]. The carbonation of concrete via atmospheric CO_2_ causes the lowering in pH [1,7,8,9]. Moreover, the Cl^−^ ions from the deicing salts or other impurities of the concrete cause impairment in the properties of passive film onto the steel rebar. The Cl^−^ ions attack locally onto the steel rebar, de-passivate the surface, and cause a pitting corrosion [10,11,12,13]. The passive film onto the steel rebar is not stable when the pH value is reduced below 9 inside the concrete [14]. Gouda [15] has described the mechanism for the breakdown of passive film onto the steel rebar in the presence of Cl^−^ ions and mentioned that once the Cl^−^/OH^−^ ratio reaches 0.6, the breakdown of the passive film arises even at the high pH. Once the corrosion phenomena start onto the steel rebar, the volume of the corrosion products increase result in cracks and spalling of the concrete.

Extensive efforts are being carried out by researchers worldwide to mitigate the corrosion of steel rebars inside the contaminated concrete environment. Different approaches such as use of corrosion resistance steel, coating, cathodic protection, polymeric coating, and using of inhibitors are being adopted. The application of the inhibitors is considered to be one of the most effective methods to reduce the corrosion of the steel rebars inside the concrete [13,16,17].

The phosphate-based compound is one of the best and effective inhibitors to mitigate the corrosion of steel rebar in chloride contaminated concrete pore solution [18] owing to its eco-friendly nature. The phosphate-based corrosion inhibitor reduces the corrosion of steel rebar in chloride contaminated environments by reducing the ingress of Cl^−^ ions and does not have any harmful effect on the concrete as well as human being [19,20,21,22,23]. However, some other researchers have used it as conversion coating and pigment to mitigate the corrosion [24,25,26,27]. The nature of phosphate-based inhibitor is not clear. However, some researchers have claimed that the phosphate-based inhibitors act as anodic, cathodic, and mixed types of inhibitor [28,29,30]. Moreover, the phosphate-based inhibitor is more environmentally friendly than the nitrite-based [31,32]. The nitrite-based and other organic inhibitors have a toxic effect either on the properties of concrete or human being [17,33,34,35].

It was reported that sodium monofluorophosphate (Na_2_PO_3_F) and Na_3_PO_4_ form a very protective passive film onto the steel rebar surface and increase the chloride threshold value when exposed in a carbonated condition [21,22,23,36,37,38]. Moreover, phosphate-based compounds have been used as a pore sealing agent to improve the corrosion resistance properties of coating. Sodium phosphate has been used to fill the pores of Al coating deposited by the arc thermal spray process, which enhances the properties of coating as well as improved the corrosion resistance at an early age of exposure but once the duration has increased, it shows a detrimental effect in artificial ocean water solution [39]. In addition, 0.1 M Ca(NO_3_)_2_ plays an important role in the improvement of corrosion resistance properties of sodium phosphate treated Al coating [40]. The post-treatment with ammonium phosphate monobasic or ammonium phosphate monobasic with 0.1 M Ca(NO_3_)_2_ has improved the morphology as well as corrosion resistance properties of Al coating compared to the coated one at longer duration of exposure in artificial ocean water [9,41].

Due to the environmental awareness, there is a demand of eco-friendly corrosion inhibitors. The ammonium phosphate monobasic is an eco-friendly, economical, and green fertilizer. It is discussed in the aforementioned paragraphs that ammonium phosphate monobasic is an excellent pore sealing agent to improve the corrosion resistance properties of Al coating. Thus, it is our prudent thought to use 1 M ammonium phosphate mono basic as inhibitor to combat the corrosion of steel rebar exposed in a concrete environment. In the present work, we have systematically studied the corrosion mitigation kinetics and mechanism of steel rebar using different amounts, i.e., 1–3 *v*/*v*% of 1 M ammonium phosphate monobasic in simulated concrete pore (SCP) solution contaminated with 3.5 wt% NaCl. To understand the corrosion kinetics and mechanism, potentiodynamic polarization, open circuit potential (OCP), and electrochemical impedance spectroscopy (EIS) methods are used with different exposure periods. The characterizations of passive film formed onto the steel rebar surface after 120 h of exposure have been carried out by scanning electron microscopy (SEM) and Raman spectroscopy.

## 2. Experimental Details

### 2.1. Materials and Methods

A 16 mm diameter and 100 mm length black oxide containing steel rebar was cut into 10 mm thickness. The black oxide was very adherent and thick thus, it was required to be removed. The black oxide was firstly removed by pickling with 10 *v*/*v*% hydrochloric acid solution up to 5 min then thoroughly washed with distilled water, rinsed with ethanol, and dried [13,14]. Thereafter, the steel rebars were mechanically abraded with emery papers from 220 to 2000 grit sizes then cloth polished using 0.5 μm particle size alumina slurry to make the surface defect free and mirror finished. The chemical compositions of the steel rebar are C = 0.240, Mn = 0.950, Cr = 0.040, Ni = 0.030, Cu = 0.020, Mo = 0.010, Sn = 0.001, Si = 0.260, P = 0.016, S = 0.008 (wt%), and Fe is remainder. Prior to the start of the electrochemical experiment, the mirror polished steel rebars were degreased with acetone.

The simulated concrete pore (SCP) solution was prepared by dissolving the analytical grade of 8.33 g/L sodium hydroxide (NaOH), 3.36 g/L potassium hydroxide (KOH), and 2 g/L calcium oxide (CaO) in distilled water using an automatic magnetic stirrer (MS300HS, MTOPS, Seoul, Korea) up to 24 h at 20 (±1) °C [42,43]. This solution simulates the concrete environment. Subsequently, the SCP solution was filtered by 5C number (110 mm) Wattman filter paper to ascertain that the insoluble CaO did not come into the solution, thereafter, 3.5 wt% NaCl was added to induce the corrosion reaction of the steel rebar. This solution is designated as SCP + 3.5 wt% NaCl (bare). The pH of this solution was measured and found to be 12.65 at 20 (±1) °C.

The inhibitor solution was prepared by dissolving the analytical grade of 1 M ammonium phosphate monobasic (NH_4_H_2_PO_4_) salt in distilled water by vigorous stirring on a magnetic stirrer for 30 min at 20 (±2) °C. The pH of this solution was found to be 3.9 at 20 (±1) °C. This solution cannot be used directly into the SCP + 3.5 wt% NaCl solution owing to the acidic nature to induce the corrosion of steel rebar. Thus, different amounts, i.e., 1, 2, and 3 *v*/*v*% of 1 M NH_4_H_2_PO_4_ were chosen to be used as inhibitor. However, the threshold pH value for the initiation of steel rebar corrosion is 9 [14]. Thus, the pH of each inhibitor solution was increased up to 9 by adding 1 M NaOH solution dropwise, thereafter, the inhibitor solutions were added in SCP + 3.5 wt% NaCl solution. After the addition of 1, 2, and 3 *v*/*v*% (pH of each solution was 9) inhibitor in SCP + 3.5 wt% NaCl solution, the pH was found to be 12.70, 12.72, and 12.73 at 20 (±1) °C, respectively. The pH of each solution was slightly increased owing to the evolution of NH_4_OH during hydrolysis of ammonium phosphate in alkaline environment.

### 2.2. Electrochemical Studies

The electrochemical studies were performed by three electrode systems where the steel rebar acted as working electrode (WE), platinum wire as counter electrode (CE), and Hg/Hg_2_Cl_2_ (saturated calomel electrode: SCE) as reference electrode (RE). The WE and RE were kept at a minimum distance to minimize the solution resistance. The exposure area of the WE was fixed at 0.78 cm^2^ for all samples. The electrochemical impedance spectroscopy (EIS) studies were performed with different exposure periods at variable frequency ranging from 100 kHz to 0.01 Hz at 10 mV sinusoidal voltage. The potentiodynamic polarization of the steel rebar samples exposed to with and without the inhibitor solution was carried out after 1 h of exposure at a 1 mV/s scan rate ranging from −0.4 to +0.8 V vs. SCE. All the electrochemical experiments were performed by VersaSTAT (Princeton Applied Research, Oak Ridge, TN, USA) potentiostat in triplicate sets of steel rebar samples at 20 (±1) °C and the average was recorded for the result. The obtained data by the potentiostat were analyzed by fitting the experimental data in a constant phase element (CPE) model using Metrohm Autolab Nova 1.10 software (Metrohm Autolab B.V., Utrecht, The Netherlands).

### 2.3. Characterization of Passive Film

The morphology of the passive film formed onto the steel rebar surface after 120 h of exposure in 3% inhibitor-added SCP + 3.5 wt% NaCl and bare solutions were characterized by scanning electron microscopy (SEM, MIRA3, TESCAN, Brno, Czech Republic) equipped with energy-dispersive X-ray spectroscopy (EDS) at 15 kV.

The nature of passive film formed onto the steel rebar surface after 120 h of exposure in 3% inhibitor-added SCP + 3.5 wt% NaCl and bare solutions were characterized by Raman spectroscopy (Horiba, LabRAM HR, Villeneuve d’Ascq, France) using He-Cd diode laser beam with a 325 nm wavelength. During the collection of Raman spectra, the laser power was kept low, i.e., 1 mW as much as possible for 10 s of incident laser light. The scans were performed from 200 to 600 cm^−1^.

## 3. Results and Discussion

### 3.1. Potentiodynamic Polarization

**Figure 1:**
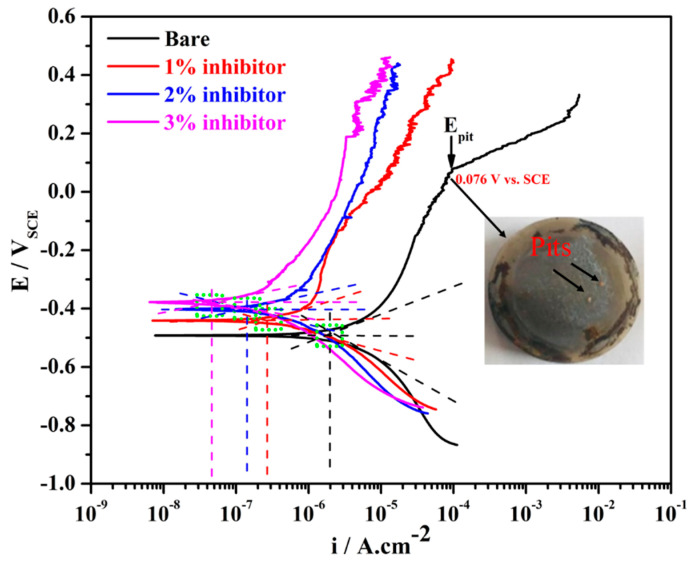
Potentiodynamic polarization plots of steel rebars after 1 h of exposure in simulated concrete pore (SCP) + 3.5 wt% NaCl solution with and without (bare) inhibitor.

The potentiodynamic polarization results of steel rebars after 1 h of exposure in different amounts of inhibitor-added SCP + 3.5 wt% NaCl and bare solutions are shown in Figure 1. From this figure, it can be seen that the steel rebar exposed to the bare solution exhibited higher cathodic as well as anodic current density compared to the inhibitor-added SCP + 3.5 wt% NaCl solutions. As the amount of ammonium phosphate monobasic inhibitor is increased in SCP + 3.5 wt% NaCl solution, the cathodic and anodic current densities are gradually decreased attributing to the formation of protective passive film onto the steel rebar surface. The steel rebar exposed to the bare solution is cathodically less polarized owing to the formation of less protective and porous passive/oxide film, resulting in higher current density [44]. However, as the inhibitor amount is increased in SCP + 3.5 wt% NaCl solution, the cathodic current density is decreased gradually. This result suggests that the steel rebar exposed to 3% inhibitor-added SCP + 3.5 wt% NaCl solution leads to form a very protective passive/oxide film thereby reducing the cathodic current density.

The steel rebars exposed to inhibitor-added SCP + 3.5 wt% NaCl solutions exhibited fluctuation in the anodic current density attributing to the formation of semi-conducting passive film where Cl^−^ ions locally attack and breakdown the passive film [45]. However, subsequently the phosphate ions (from ammonium phosphate monobasic) help in the formation of passive film. It can be seen from Figure 1 that the steel rebars exposed to 1% inhibitor-added SCP + 3.5 wt% NaCl solution started to breakdown the passive film at −0.008 V vs. SCE on 8.90 µA·cm^−2^, whereas, 2 and 3% inhibitor-added solutions at 0.142 V vs. SCE on 7.70 µA·cm^−2^ and 0.186 V vs. SCE on 3.39 µA·cm^−2^, respectively. As the inhibitor amounts are increased, the breakdown potentials of exposed steel rebars have shifted towards nobler direction and anodic current density decreased owing to the formation of passive film. This result suggests that the higher amount of inhibitor strengthens the passive film. However, the steel rebar exposed to the bare solution shows the pitting potential (*E_pit_*) at 0.076 V vs. SCE where the anodic current density is increased abruptly. It is attributed to the formation of less protective passive film where Cl^−^ ions significantly breakdown the film and lead to form pits onto the steel rebar surface, as can be seen in the inset of Figure 1.

The electrochemical parameters of steel rebars for 1 h of exposure to the bare and inhibitor-added SCP + 3.5 wt% solutions after fitting of potentiodynamic polarization plots in Tafel regions are shown in Table 1 with error values. As the amount of inhibitor is increased in SCP + 3.5 wt% NaCl solution, the corrosion potential (*E_corr_*) of steel rebars is shifted towards positive (nobler) direction owing to the formation of uniform passive film. It is reported earlier that if the steel rebar exposed to the inhibitor containing solution shows *E_corr_* ≥ −0.085 V vs. SCE compared to the bare solution, then such type of inhibitor works as anodic inhibitor [14,46,47]. In the present study, the steel rebars exposed to 2 and 3% inhibitor-added SCP + 3.5 wt% NaCl solutions exhibited *E_corr_* ≥ −0.085 V vs. SCE compared to the steel rebar exposed to the bare solution. Thus, it can be said that ammonium phosphate monobasic acts as an anodic inhibitor, whereas, it is also required in higher amount to mitigate the corrosion of steel rebars.

From Table 1, it can be seen that the corrosion current density (*i_corr_*) of the steel rebars exposed to bare, 1, 2, and 3% inhibitor-added SCP + 3.5 wt% NaCl solutions is found to be 2.52, 0.33, 0.18, and 0.06 µA·cm^−2^, respectively. On the basis of *i_corr_* value, the corrosion rate can be calculated as follows [48]:

(1) $$Corrosion\;rate\;(\mu m\cdot {\mathrm{year}}^{-1})=\frac{3.27\times i_{corr}\times E.W.}{d}$$

where *i_corr_* is the corrosion current density (µA·cm^−2^) obtained by dividing the total surface area of the working electrode, i.e., 0.78 cm^2^ in the current density. *E.W.* represents the equivalent weight (g. mole^−1^) and *d* is the density (g·cm^−3^) of iron. From Table 1, it can be seen that the steel rebar exposed to 3% inhibitor-added SCP + 3.5 wt% NaCl solution exhibited the lowest corrosion rate, i.e., 0.70 µm·year^−1^ while the highest to be found in bare, i.e., 29.28 µm·year^−1^. The highest corrosion rate of steel rebar exposed to the bare solution owing to the aggressiveness of Cl^−^ ions to induce the corrosion reaction. The efficiency of the inhibitors can be obtained from *i_corr_* values by [49,50]:

(2) $$\mathrm{Efficiency}\;(\%)\;=\;\left[1-\frac{i_{corr\;(with\;inhibitor)}}{i_{corr\;(bare)}}\right]\times 100$$

The corrosion inhibition efficiencies of steel rebars exposed to 1, 2, and 3% inhibitor-added SCP + 3.5 wt% NaCl solutions are found to be 86.90, 92.86, and 97.62%, respectively after 1 h of exposure.

### 3.2. Adsorption Isotherm

**Figure 2:**
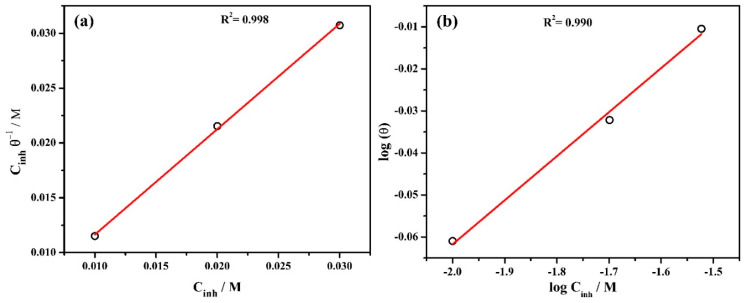
Inhibitor adsorption isotherm (**a**) Langmuir and (**b**) Freundlich at 20 °C.

The adsorption isotherm of inhibitor onto the steel rebar surface is described by the corrosion efficiency calculated from potentiodynamic polarization plots and the adsorption parameters are shown in Table 2. The Langmuir adsorption isotherm is calculated as [51]:

(3) $$\frac{C_{inh}}{\theta }=\frac{1}{K_{ads}}+C_{inh}$$

where *C_inh_* is the concentration of inhibitor in mol/L and *θ* refers to the surface coverage defined from the inhibitor efficiency (%). *K_ads_* is the equilibrium adsorption constant. The *θ* of 1, 2, and 3% inhibitor is found to be 0.8690, 0.9286, and 0.9762 (Table 2), respectively. The Langmuir adsorption isotherm is shown in Figure 2a where $\frac{C_{inh}}{\theta }$ versus *C_inh_* represents a straight line and the slope is found to be 0.96 which is close to 1. Thus, this finding suggests that the Langmuir adsorption isotherm is appropriate for the adsorption behavior of inhibitor molecules onto the steel rebar surface [52].

Moreover, the inhibitor adsorption isotherm is also calculated using the Freundlich equation [53]:

(4) $$\log(\theta )=\log K_{F}+\frac{1}{n}\log C_{inh}$$

where *K_F_* and $\frac{1}{n}$ are the Freundlich constant and slope, respectively. If $\frac{1}{n}$ value is close to zero then the surface is heterogeneous, whereas, 0 to 1 implies a favorable adsorption condition. In the present study, $\frac{1}{n}$ is found to be 0.104 which confirms the favorable adsorption condition [53]. The regression coefficient (R^2^) is found to be 0.990 (Figure 2b) which indicates that the adsorption of inhibitor molecules onto the steel rebar surface is consistent.

The standard adsorption free energy is calculated by [53]:

(5) $$K_{ads}=\frac{1}{55.5}\exp\left(-\frac{\Delta G_{ads}^{0}}{RT}\right)$$

where *R* is the molar gas constant (J/K/mol), *T* is the absolute temperature (K), and 55.5 is the concentration of water in the solution expressed in moles. The $\Delta G_{ads}^{0}$ value is found to be −24.92 and −10.61 kJ/mol for Langmuir and Freundlich methods (Table 2), respectively. The negative value of $\Delta G_{ads}^{0}$ indicates the spontaneous adsorption and stability of the adsorbed film onto the steel rebar surface. The $\Delta G_{ads}^{0}$ calculated by the Langmuir adsorption isotherm indicates that the ammonium phosphate monobasic inhibitor follows the physisorption and chemisorption together [54,55,56,57] while the Freundlich result suggests the physisorption isotherm [58].

### 3.3. Open Circuit Potential (OCP) with Exposure Periods

**Figure 3:**
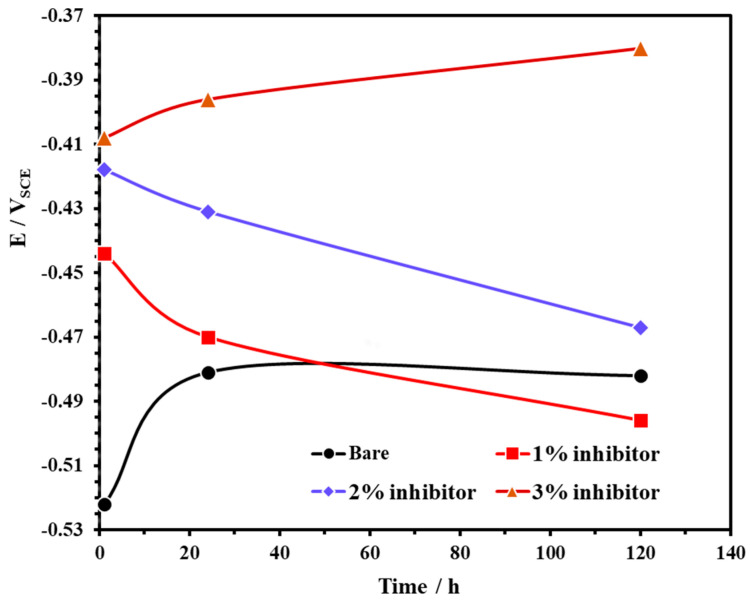
The open circuit potential (OCP) plots of steel rebar exposed to SCP + 3.5 wt% NaCl solution with and without inhibitor.

The open circuit potential (OCP) of steel rebars exposed to bare and inhibitor-added SCP + 3.5 wt% NaCl solutions are shown in Figure 3. From this figure, it can be observed that the steel rebars exposed to the bare and 3% inhibitor-added SCP + 3.5 wt% NaCl solutions exhibited ennobling in OCP while 1% and 2% inhibitor-added solutions are shifted towards active directions. The OCP of steel rebar samples exposed to the bare solution showed at −0.522 V vs. SCE for 1 h while 1, 2, and 3% inhibitor-added solutions at −0.444, −0.418, and −0.408 V vs. SCE, respectively. However, once the exposure periods are extended, the OCP of the steel rebars exposed to 1 and 2% inhibitor-added SCP + 3.5 wt% NaCl solutions are gradually shifted towards active directions which reveal the breakdown of passive film attributed to the acidification as well as the effect of Cl^−^ ions. The phosphate ions in 1 and 2% inhibitor-added solutions are not significant to retain the passivation while Cl^−^ (from solution) and H^+^ (from ammonium phosphate monobasic) ions induce the corrosion reaction. Thus, the active OCP is observed with exposure periods (Figure 3). However, the steel rebars exposed to 3% inhibitor-added SCP + 3.5 wt% NaCl solution contains a significant amount of H^+^ and phosphate ions. H^+^ and Cl^−^ ions induce the corrosion reaction while phosphate ions transform the unstable corrosion products into a stable iron phosphate as passive film, respectively. Thus, it is found that as the exposure periods are increased, the OCP is shifted towards a nobler/positive direction. The steel rebars exposed to the bare solution initially deteriorated owing to the Cl^−^ ions and formed the corrosion products, thus, the active OCP is observed for 1 h of exposure. However, once the exposure period reaches from 24 to 120 h, these corrosion products deposit onto the steel rebar surface thereby shifting the OCP towards a positive (nobler) direction as observed in Figure 3.

### 3.4. Electrochemical Impedance Spectroscopy (EIS) Studies with Exposure Periods

**Figure 4:**
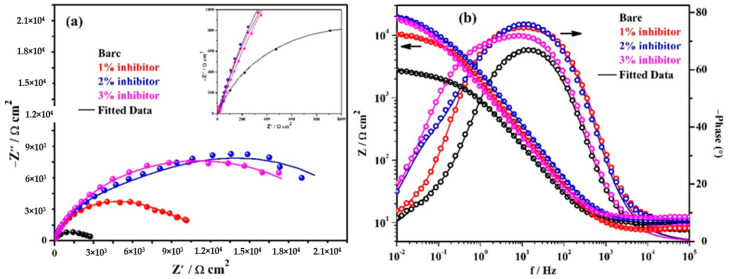
Electrochemical impedance spectroscopy (EIS) plots. (**a**) Complex plane impedance and (**b**) Bode spectra of steel rebars after 1 h of exposure in SCP + 3.5 wt% NaCl solution with and without inhibitor.

The EIS results of triplicate steel rebar samples and the average values of the obtained results after 1 h of exposure in bare and inhibitor-added SCP + 3.5 wt% NaCl solutions are shown in Figure 4. The surface characteristics of steel rebar can be seen at higher studied frequencies, i.e., 10^5^ to 10^2^ Hz, as shown in the inset of Figure 4a. From Figure 4a, it can be seen that as the amount of inhibitor is increased in SCP + 3.5 wt% NaCl solution, the magnitude of complex plane impedance plots is increased after 1 h of exposure. It is attributed to the formation and strengthening of passive film which cover all over the steel rebar surface. However, the steel rebars exposed to the bare solution exhibit lowest in magnitude of complex plane impedance owing to the initiation of corrosion reaction by Cl^−^ ions onto the surface. Moreover, after exposure of steel rebars in bare and inhibitor added solutions exhibited homogenous and porous corrosion, products attributed to the reaction at steel surface/solution interface. It seems that at short-term of exposure, porous corrosion products could form a double layer which corroborate with the electrochemical behavior of the examined samples as shown in the inset of Figure 4a. In distinctive immersion periods, the porous corrosion products seem to be associated with planar electrode (polished steel rebar) behavior to prescribe the corrosion kinetic. With the increase of both the immersion period and inhibition content, it is speculated that the planar electrode (polished steel rebar) becomes prevalent.

The modulus-frequency Bode plots of the steel rebar samples after 1 h of exposure are shown in Figure 4b. The steel rebars exposed to inhibitor-added SCP + 3.5 wt% NaCl solution have shown higher total impedance at 0.01 Hz owing to the formation of protective passive film compared to bare solution after 1 h of exposure. As the concentration of inhibitor in SCP + 3.5 wt% NaCl solution is increased, the total impedance values of the steel rebars are increased gradually at the lowest studied frequency. The total impedance of steel rebars exposed to 1% inhibitor-added SCP + 3.5 wt% NaCl solution are 4-fold higher, whereas, 2 and 3% inhibitor-containing solutions are approximately 7-fold compared to bare solution.

The phase-frequency Bode plots of the steel rebars after 1 h of exposure are shown in Figure 4b. From this figure, it can be seen that the phase-angle maxima of the steel rebars exposed to inhibitor-added SCP + 3.5 wt% NaCl solutions are shifted to approximately −76° owing to the greater coverage of surface area by the ammonium phosphate monobasic inhibitor. However, the phase angle maxima shift of the steel rebar exposed to 3% inhibitor-added solution is found to be −70° from 24 to 0.90 Hz whereas 1 and 2% inhibitors are found to be at −74° on 11 Hz and −76° from 32 to 3.5 Hz, respectively. Moreover, the phase angle maxima of the steel rebar exposed to 1% inhibitor-added solution is asymmetric and sharp. This result suggests that the lower amount of inhibitor can provide protection to the steel rebar but the surface coverage is not uniform. Thus, the impedance of the steel rebar exposed to 1% inhibitor-added solution is lower than 2 and 3% inhibitor-added solutions. The phase angle maxima of the steel rebars exposed to bare solution is found to be around −67° from 33 to 6 Hz. The lowest in phase angle maxima of the steel rebar exposed to bare solution reveals the deterioration as observed in total impedance where its value is minimum (Figure 4b). The phase angle maxima shift of the steel rebars at the lowest studied frequency, i.e., 0.01 Hz exposed to bare, 1, 2, and 3% inhibitor-added solutions are found to be at −8, −10.5, −17.5, and −20.5°, respectively. This result suggests that as the amount of inhibitor is increased, the phase angle maxima at 0.01 Hz is increased which reveals the passive behavior of steel rebars by the formation of protective film.

**Figure 5:**
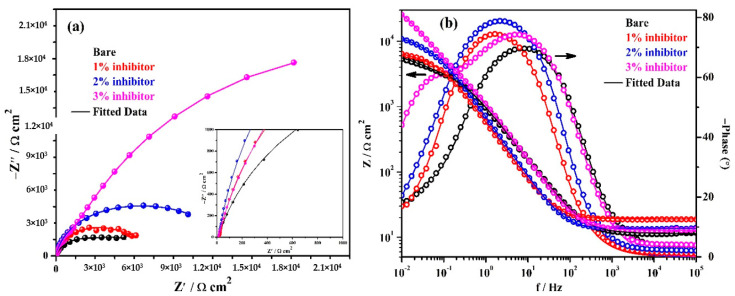
EIS plots. (**a**) Complex plane impedance and (**b**) Bode spectra of steel rebars after 24 h of exposure in SCP + 3.5 wt% NaCl solution with and without inhibitor.

The EIS studies are performed at prolonged exposure periods. The complex plane impedance plots of the steel rebars exposed to bare and inhibitor-added SCP + 3.5 wt% NaCl solutions after 24 h of exposure are shown in Figure 5a. It can be seen from this figure that the steel rebars exposed to bare and 3% inhibitor-added solutions exhibited an increase in magnitude of complex plane impedance compared to 1 h of exposure, whereas, 1 and 2% inhibitor-added solutions showed a decrease in magnitude. The decrease in magnitude after 24 h of exposure is owing to the acidification of solution and attack of Cl^−^ ions. However, the steel rebar exposed to 1 and 2% inhibitor-added solutions contain less amount of phosphate ions to form a protective passive film. Moreover, Cl^−^ and H^+^ ions are dominated, therefore the deterioration of steel rebars is observed after 24 h of exposure compared to 1 h. In addition, the steel rebar exposed to bare solution simultaneously form the corrosion products owing to the presence of a high amount of Cl^−^ ions. Consequently, once corrosion has started, the corrosion product instantaneously starts to deposit onto the steel rebar surface after 24 h of exposure which provides barrier protection from ingress of Cl^−^ ions, thus, OCP is shifted towards a nobler direction (Figure 3). However, the chloride bearing complex porous oxide (formed up to 24 h of exposure), i.e., Fe(OH)_x_Cl_y_O_z_ films might influence the corrosion reaction at prolonged exposure periods. In this condition, ammonium phosphate acts as a rust converter rather to adsorb onto the steel surface. Thus, it is required to investigate the corrosion mechanism at longer duration of exposure using EIS. In subsequent paragraphs, discussion has been illustrated on the corrosion mechanism after 120 h of exposure. However, the electrochemical reactions that occur at the steel rebar/solution interface in the presence of inhibitor after 24 h of exposure are described as:

(6) $$\mathrm{Fe}+\frac{1}{2}O_{2}+H_{2}O\to {\mathrm{Fe}(\mathrm{OH})}_{2}$$

Initially, once steel (Fe) is exposed in the solution simultaneously it forms Fe(OH)_2_ as a corrosion product (Equation (6)). Generally, this corrosion product (Equation (6)) is very unstable and soluble [59]. Therefore, there is a possibility that Fe(OH)_2_ transforms into another oxide by reacting with the inhibitor rather than adsorption onto the steel rebar surface. In the present study, ammonium phosphate monobasic (NH_4_H_2_PO_4_) as the inhibitor reacts with water (H_2_O) molecules and forms phosphoric acid (H_3_PO_4_) and ammonium hydroxide (NH_4_OH) as shown in Equation (7). Due to the liberation of NH_4_OH (Equation (7)) during the preparation of solution, initially the pH of 3% inhibitor-added solution was alkaline (pH = 12.73 at 20 °C). However, once the exposure periods are increased up to 24 h, then most of the NH_4_OH has liberated and H_3_PO_4_ remains in the solution, thus the pH is reduced to 12.40 at 20 °C. Simultaneously, H^+^ (from H_3_PO_4_) and Cl^−^ ions (from NaCl) cause the corrosion of steel rebars to result in the formation of a significant volume of Fe(OH)_2_ as corrosion products. The phosphoric acid (H_3_PO_4_) again reacts with Fe(OH)_2_ and forms tertiary iron phosphate (FePO_4_) as described in Equation (8). The FePO_4_ is thermodynamically very stable and sparingly soluble [60]. Thus, the magnitude in complex plane impedance plots of the steel rebars after 24 h of exposure in 3% inhibitor-added solution exhibit as highest compared to the other samples. While in the case of 1 and 2% inhibitor-added solutions, phosphate ions are not optimum thus, H^+^ and Cl^−^ ions dominate to induce the corrosion of steel rebars rather than the transformation of Fe(OH)_2_ into FePO_4_:

(7) $${\mathrm{NH}}_{4}H_{2}{\mathrm{PO}}_{4}+H_{2}O\to H_{3}{\mathrm{PO}}_{4}+{\mathrm{NH}}_{4}\mathrm{OH}$$

(8) $$\begin{array}{l} {\mathrm{Fe}(\mathrm{OH})}_{2}+H_{3}{\mathrm{PO}}_{4}+e^{-}\to {\mathrm{FePO}}_{4}+2H_{2}O+H^{+}\\ \;↓\\ \;(\mathrm{tertiary}\;\mathrm{iron}\;\mathrm{phosphate}) \end{array}$$

The modulus-frequency Bode plots of samples after 24 h of exposure are shown in Figure 5b. It can be seen that the total impedance at 0.01 Hz of steel rebars exposed to 1 and 2% inhibitor-added solutions are decreased whereas bare and 3% inhibitor-added solutions are increased after 24 h of exposure compared to 1 h. However, the total impedance values of the steel rebars exposed to 1 and 2% inhibitor-added solutions are higher compared to the bare solution. The steel rebars exposed to 1, 2, and 3% inhibitor-added solutions exhibit approximately 1-, 2-, and 5-fold higher in total impedance values compared to the bare solution after 24 h of exposure. The decrease in total impedance of the steel rebars exposed to 1 and 2% inhibitor-added SCP + 3.5 wt% NaCl solutions after 24 h of exposure are attributed the deterioration phenomena. In these cases, H^+^ and Cl^−^ ions induce the corrosion of steel rebars that result in the formation of unstable corrosion products which dissolve in the solution rather than deposit onto the steel rebar surface. The phosphate ions are not significant in 1 and 2% inhibitor-added solutions which play a vital role in the transformation of unstable corrosion products into a stable one. Thus, the decrease in impedance is observed after 24 h of exposure compared to 1 h. In addition, the steel rebar exposed to the bare solution initially corrodes and forms some corrosion products which causes hindrance in the ingress of aggressive ions. Thus, after 24 h of exposure, the total impedance values are increased compared to 1 h. Moreover, the 3% inhibitor-added solution has the highest amount of H^+^ as well as phosphate ions. Cl^−^ and H^+^ ions induce the corrosion reaction of steel rebars but subsequently a significant amount of phosphate ions transform the Fe(OH)_2_ into FePO_4_ as described in Equation (8). Thus, the increase in total impedance values are observed after 24 h of exposure. This result suggests that ammonium phosphate monobasic firstly gets adsorbed onto the steel rebar surface up to 1 h but as the exposure periods are extended, due to the presence of H^+^ and Cl^−^ ions, corrosion products have started to form. Then, at this stage, the inhibitor acts as a rust converter to transform the Fe(OH)_2_ into FePO_4_ rather than to adsorb onto the steel rebar surface. If the formation of corrosion products are significant due to the breakdown of passive film by Cl^−^ and H^+^ ions, then the transformation of unstable corrosion products, i.e., Fe(OH)_2_ would be higher into the stable one in the presence of inhibitor which cover all over the steel rebar surface. Thus, corrosion studies at prolonged exposure periods are required to understand the corrosion mechanism and transformation of unstable corrosion products into a stable one.

The phase-frequency Bode plots of samples after 24 h of exposure are shown in Figure 5b. It can be seen from this figure that the steel rebars exposed in the bare solution exhibit the phase angle maxima at −70° on 8 Hz that is slightly higher than 1 h of exposure but possesses capacitive properties of the passive film/corrosion products. The phase angle maxima of steel rebars exposed to 1 and 2% inhibitor-added SCP + 3.5 wt% NaCl solutions are reduced but shifted towards low frequencies while the 3% inhibitor-added solution shows an increase in values compared to earlier exposure periods. The shifting of phase angle maxima at a low angle is attributed to the deterioration of the steel rebars. The steel rebars exposed to 3% inhibitor-added solution shows two distinct capacitive loops at −74° and −61° on 5 and 0.1 Hz, respectively. However, after 1 h of exposure, it is only one loop but broadening is observed at −70° from 24 to 0.90 Hz (Figure 4b). The presence of two capacitive loops indicate the formation of very protective passive/oxide film onto the steel rebar. The shifting of phase angle maxima at −44° on 0.01 Hz reveal that the steel surface is being protected by passive/oxide film after the transformation of Fe(OH)_2_ into FePO_4_.

**Figure 6:**
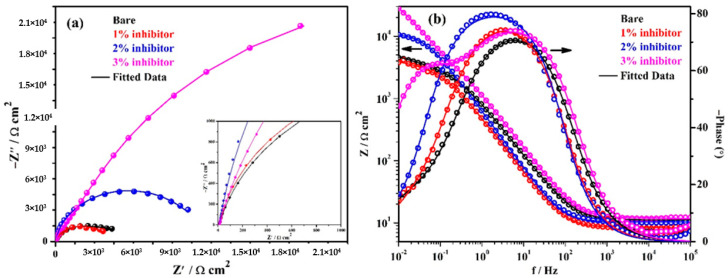
EIS plots. (**a**) Complex plane impedance and (**b**) Bode spectra of steel rebars after 120 h of exposure in SCP + 3.5 wt% NaCl solution with and without inhibitor.

The EIS plots are performed at continuous exposure of steel rebars in different solutions to study the corrosion mechanism of inhibitor and results are shown in Figure 6. The complex plane impedance plots of steel rebars after 120 h of exposure in bare and inhibitor-added SCP + 3.5 wt% NaCl solutions are shown in Figure 6a. From this figure, it is depicted that the magnitude in complex plane impedance of the steel rebars exposed to 1% inhibitor-added solution is reduced significantly compared to earlier exposure periods and even it is less than the sample exposed to the bare solution. It is attributed to the severe corrosion of steel rebar surface owing to the cumulative effect of H^+^ and Cl^−^ ions. Moreover, it can be seen that the steel rebars exposed to the bare solution show reduction in complex plane impedance magnitude compared to 24 h of exposure. It is attributed to the formation of unstable oxide film where Cl^−^ ions easily penetrate and reach towards the steel rebar surface. However, the steel rebars exposed to 2% inhibitor-added solution also show reduction in complex plane impedance magnitude after 120 h of exposure compared to 24 h but greater than the bare solution. In this case, H^+^ and Cl^−^ ions simultaneously induce the deterioration of the steel rebars and help dissolve the unstable corrosion products. In addition, the steel rebars exposed to 3% inhibitor-added solution exhibit slightly higher in Z_real_ and −Z_imag._ magnitude after 120 h compared to 24 h which reveal the highest corrosion resistance properties. It is attributed to the formation of stable passive/oxide film onto the steel rebar surface. This result suggests that 24 h is the incubation period which is required to transform the unstable corrosion products into a stable one. In this case, the corrosion products which have formed up to 24 h of exposure, mostly have been transformed into a stable one and cover all over the steel rebar surface uniformly.

The modulus-frequency Bode plots of the steel rebars after 120 h of exposure are shown in Figure 6b. It is depicted from this figure that the total impedance of the steel rebars exposed to the bare, 1, and 2% inhibitor-added solutions are decreased compared to 24 h of exposure, whereas the 3% inhibitor-added solution exhibits an increase in its value. The decrease in total impedance of the steel rebar exposed to bare solution is attributed to the deterioration where Cl^−^ ions from the solution penetrate through the corrosion products, i.e., Fe(OH)_2_ and reduce the localized pH as described below and attack onto the steel surface. Thus, the total impedance values are decreased after 120 h of exposure [6]:

(9) $${\mathrm{Fe}(\mathrm{OH})}_{2}+2{\mathrm{Cl}}^{-}\to {\mathrm{FeOCl}}_{2}^{--}+H_{2}O$$

(10) $${\mathrm{FeOCl}}_{2}^{--}+H_{2}O\to {\mathrm{FeCl}}_{2}+2{\mathrm{OH}}^{-}$$

(11) $${\mathrm{FeCl}}_{2}+2H_{2}O\to {\mathrm{Fe}(\mathrm{OH})}_{2}+2\mathrm{HCl}$$

Fe(OH)_2_ reacts with Cl^−^ ions and forms FeOCl_2_ (Equation (9)) which again reacts with water (H_2_O) molecule and forms FeCl_2_ (Equation (10)). This FeCl_2_ again reacts with H_2_O and forms Fe(OH)_2_ as well as HCl (hydrochloric acid) in Equation (11). The HCl locally reduces the pH to induce the corrosion reaction [61] thus, a lower impedance value is observed (Figure 6b).

The phase-frequency Bode plots of steel rebars after 120 h of exposure in solutions are shown in Figure 6b. From this figure, it can be seen that the steel rebars exposed to the bare solution exhibit an asymmetric peak at −70° on 6 Hz and are identical as obtained after 24 h of exposure. However, the steel rebars exposed to 1% inhibitor-added solution show the phase angle maxima at −15° as identical obtained after 24 h of exposure but shifted towards a lower frequency. The steel rebars exposed to 2% inhibitor-added solution exhibit the shifting of phase angle maxima at −79° on 1 Hz. Therefore, the steel rebars exhibit a reduction in the total impedance values compared to 24 h of exposure. Moreover, it is observed that the steel rebars exposed to 2% inhibitor-added solution exhibit the phase angle maxima shift at a higher angle compared to bare and 1% inhibitor-added solutions which reveal the stability of the passive/oxide film. The steel rebars exposed to 3% inhibitor-added solution indicate the stability and strengthening of passive/oxide film after 120 h of exposure because there is no change in the phase angle maxima shift compared to 24 h. This result indicates that the passive/oxide film is uniformly formed after 24 h but it becomes strengthened once the exposure periods are increased up to 120 h. It is attributed to the formation of FePO_4_ after 24 h of exposure. Thus, it is concluded that once the exposure periods are increased, the passive/oxide film is strengthened onto the steel rebar surface.

**Figure 7:**
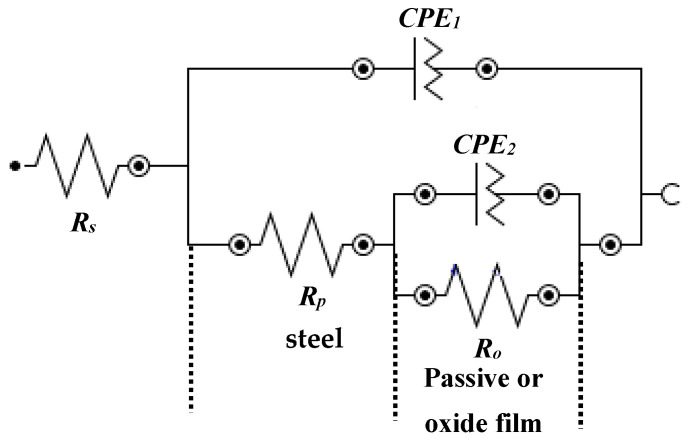
EEC of steel rebar samples exposed in 3.5 wt% NaCl contaminated concrete pore solution with and without (bare) inhibitor.

The fitting of EIS plots in suitable electrical equivalent circuit (EEC) are shown in Figure 7. This EEC is fitted where the corrosion reaction occurs at steel rebar/solution and passive or oxide film/solution interface [49,62,63,64]. It can be seen from Figure 7 that this EEC contains a two times constant. The first time constant refers to the corrosion reaction at steel/solution interface in higher frequency domain and another at passive or oxide film/solution interface in lower frequency domain due to the corrosion in bare and formation of passive or oxide film in the presence of inhibitor-added solutions, respectively. In this EEC (Figure 7), the first time constant contains the solution resistance (*R_s_*) that is combined in a series with polarization resistance (*R_p_*) and constant phase element (CPE1). *R_p_* and CPE1 are associated parallel to each other. However, once the corrosion reaction occurs onto the steel rebar surface with and without inhibitor, the passive or oxide film is being formed and subsequently another resistance is involved. Thus, another time constant is fitted. The second time constant is attributed to the resistance of passive/or oxide film (*R_o_*) which is also called charge transfer resistance. This *R_o_* is parallel with the CPE of corrosion/passive film (CPE2).

The chi-square (χ^2^) values of each fitting for different duration of exposure are obtained between 10^−2^ to 10^−3^ using the Metrohm Autolab Nova 1.10 software. The χ^2^ values indicate the best quality fitting with the experimental data [65,66,67,68,69,70]. The electrochemical parameters after fitting of EIS plots in a suitable EEC with exposure periods and respective error values are shown in Table 3. From this table, it can be seen that the *R_s_* values of the all samples are found to be between 10–18 Ω cm^2^. It means there is no significant role of *R_s_* in total impedance. The *R_p_* of steel rebar exposed to the bare solution is found to be the lowest, i.e., 1.96 (±0.15) kΩ cm^2^ among all samples, attributed to the initial corrosion of steel rebar by the attack of Cl^−^ ions, whereas, inhibitor-added solutions exhibit higher in their values. As the amount of inhibitor is increased, the *R_p_* value of steel rebar is increased gradually up to 1 h of exposure. As the exposure periods are extended from 1 to 24 h, the *R_p_* values are decreased for the steel rebars exposed to 1 and 2% inhibitor-added solutions, while bare and 3% inhibitor-added solutions show an increase in values. However, as the exposure periods of steel rebar exposed to the bare solution are extended up to 120 h, the *R_p_* value is decreases owing to the attack of Cl^−^ ions onto the steel rebar surface. On the other hand, the steel rebar exposed to 1 and 2% inhibitor-added solution contain a high amount of H^+^ as well as Cl^−^ ions. The Cl^−^ ions synergistically influence the corrosion, while H^+^ dissolve the corrosion products up to 120 h, thus, the *R_p_* value is gradually decreased. In addition, when the steel rebars are exposed to 3% inhibitor-added solution, the *R_p_* value is gradually increased up to 12.51 (±0.75) kΩ cm^2^ after 120 h of exposure which is the highest among all samples.

The CPE coefficient (*Q*_1_) and exponent (*n*_1_) of the first time constant show that after 1 h of exposure to the bare solution, the steel rebar exhibits the highest and lowest values compared to the inhibitor-added solutions, respectively. The *n*_1_ of the steel rebar exposed to the bare solution is found to be the lowest compared to others after 1 h of exposure. As the exposure periods are extended up to 120 h, the *Q*_1_ values are gradually increased and *n*_1_ values are decreased simultaneously for the steel rebars exposed to 1 and 2% inhibitor-added solutions. It is attributed to the corrosion phenomena and formation of unstable corrosion products/passive film onto the steel rebar surface. However, the steel rebar exposed to the bare solution shows a decrease in the *Q*_1_ value and increase in the *n*_1_ value up to 24 h of exposure, attributed to the deposition of corrosion products onto the steel rebar surface but, once the exposure period is extended up to 120 h, the *Q*_1_ value is increased to 23.8 × 10^−5^ (±2.09) Ω^−1^·cm^−2^·s^−n^ and *n*_1_ value is decreased to 0.76 (±0.02). It can be seen from Table 3 that *Q*_1_ and *n*_1_ values of the steel rebar exposed to 3% inhibitor-added solution are gradually decreased and increased with exposure periods, respectively up to 120 h of exposure. The *n*_1_ value indicates that the surface of steel rebar becomes homogeneous and uniform, thus, it shows the highest *R_p_* value.

The nature of oxide/passive film formed during the corrosion of steel rebars surface exposed to bare and inhibitor-added SCP + 3.5 wt% NaCl solutions can be described on the basis of obtained results as shown in Table 3. The *R_o_* and *n*_2_ values of the steel rebar exposed to the bare solution are found to be the lowest after 1 h of exposure, therefore, *Q*_2_ is the highest. While as the amount of inhibitor is increased, the *R_o_* and *n*_2_ values are increased as well as *Q*_2_ is decreased after 1 h of exposure. However, as the exposure periods are extended up to 120 h, the *R_o_* and *n*_2_ values are decreased and *Q*_2_ is increased for the steel rebars exposed to 1 and 2% inhibitor-added solutions. The steel rebar exposed to bare and 3% inhibitor-added SCP + 3.5 wt% NaCl solutions exhibit an increase in the *R_o_* values with the exposure period. The *n*_2_ value of steel rebar is found to be 0.89 (±0.03) after 120 h of exposure in 3% inhibitor-added solution which reveals that corrosion products/passive film is homogeneous and regularly deposited all over the surface.

The efficiency (%) of inhibitor can be calculated on the basis of *R_o_* obtained from fitting of EIS plots in suitable EEC by [64]:

(12) $$\mathrm{Efficiency}\;(\%)=\left[1-\frac{R_{o\;(bare)}}{R_{o\;(with\;inhibitor)}}\right]\times 100$$

where $R_{o\;\left(bare\right)}$ and $R_{o\;\left(with\;inhibitor\right)}$ are passive/oxide film resistance of steel rebars exposed to bare and inhibitor-added SCP + 3.5 wt% NaCl solutions, respectively.

The inhibitor efficiencies are shown in Table 3. It can be seen that the steel rebars exposed to 2 and 3% inhibitor-added solutions exhibit around 90.44% (±6.91) efficiency while 1% inhibitor-added solution shows 66.67% (±4.00) after 1 h of exposure. The efficiency values of inhibitor calculated by *R_o_* and *i_corr_* (Table 1) are little different owing to the experimental procedure. As the exposure periods are increased, the efficiency of 1% inhibitor is decreased drastically and found to be around −14% after 120 h. In this case, the phosphate ions are not significant to transform the unstable corrosion products into a stable one rather than deterioration. Moreover, the 2% inhibitor exhibits around 37% efficiency after 120 h of exposure. This result suggests that the 2% inhibitor is unable to mitigate the corrosion of steel rebar significantly when exposed to SCP+3.5 wt% NaCl solution owing to the less amount of phosphate ions. However, the 3% inhibitor contains a high amount of H^+^ as well as phosphate ions. The H^+^ and Cl^−^ ions induce the corrosion of steel rebars but simultaneously phosphate ions transform the corrosion products into a stable FePO_4_ and cover all over the surface. Thus, it maintains around 83% efficiency up to 120 h of exposure.

### 3.5. Nature of Corrosion Products/Passive Films Formed after 120 h of Exposure

#### 3.5.1. Scanning Electron Microscopy (SEM)

**Figure 8:**
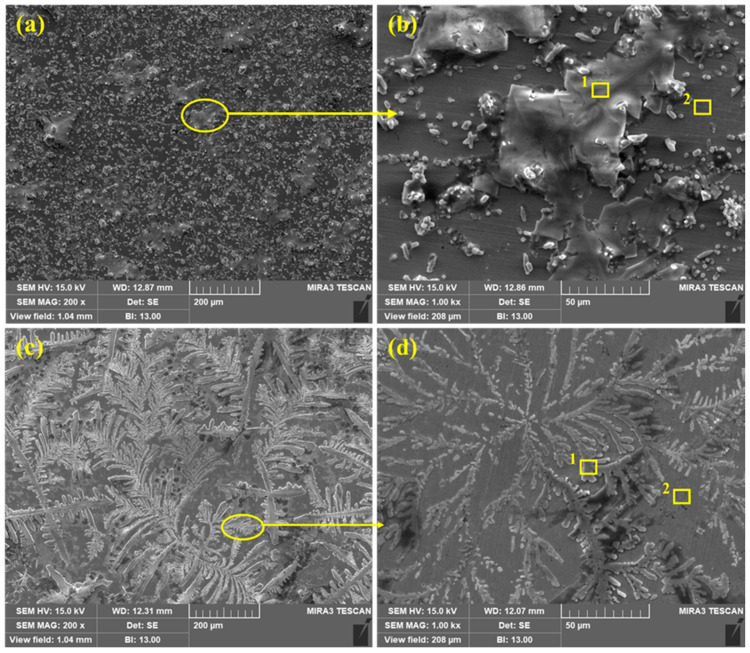
SEM of steel rebar after 120 h of exposure in bare ((**a**) at 200× and (**b**) at 1000×) and 3% inhibitor- ((**c**) at 200× and (**d**) at 1000×) added 3.5% NaCl contaminated concrete pore solutions.

The SEM of corrosion products/passive films formed onto the steel rebar surface after 120 h of exposure to bare and 3% inhibitor-containing SCP + 3.5 wt% NaCl solutions are shown in Figure 8. From Figure 8a,b, it can be seen that the steel rebar exposed to the bare solution exhibits agglomerated corrosion products with uneven morphology as well as at some places localized corrosions are observed on low magnification (Figure 8a). The agglomeration of corrosion products occurs at the cathodic site of steel rebar surface whereas Cl^−^ ions attack severely at the anodic site and form heavy corrosion products. It can be seen that corrosion products are not uniform thus, there is probability that Cl^−^ ions can attack locally to induce the corrosion reactions. Therefore, lower values in *R_p_* and *R_o_* are found after 120 h of exposure. From Figure 8b at 1000×, uneven morphology may be observed where a big part (agglomerated portion) contains defects and cracks which reveal the higher values in corrosion rate. The corrosion products are scattered to induce the corrosion phenomena. The Cl^−^ ions locally attack and enhance the corrosion reaction at longer duration of exposure.

Once the 3% inhibitor is added in SCP + 3.5 wt% NaCl solution, the morphology of the steel rebar surface becomes uniform and covers all over the surface by dendrites (Figure 8c,d) after 120 h of exposure. The dendritic structures are uniformly grown and cover the surface. The fully grown dendrites start to spread and become thick as observed in Figure 8d at 1000×. It means that the passive film strengthens with exposure periods. From Figure 8c, it can be seen that if time is extended beyond 120 h, there is a possibility that the passive film becomes dense. Therefore, the steel rebar exposed to 3% inhibitor-added SCP + 3.5 wt% NaCl solution shows highest *R_p_* and *R_o_* values after 120 h. It is attributed to the transformation of unstable corrosion products into a stable one, i.e., FePO_4_ and covers all over the surface.

The EDS analysis of the corrosion products/passive films formed onto the steel rebar surface after 120 h of exposure in bare and 3% inhibitor-containing solutions are shown in Table 4. The EDS was taken on two points at 1000× as shown in Figure 8b,d for the steel rebars exposed to bare and 3% inhibitor-containing solutions, respectively. The steel rebar exposed to the bare solution (Figure 8b) mark as 1 exhibited a high amount of Na and Cl attributed to the composition of SCP solution which contain NaOH and 3.5 wt% NaCl. Na is present in NaOH and NaCl, thus, it is found in a greater amount than Cl. The presence of K and Ca are attributed to the pore solution which contain KOH and CaO. O is found to be the least. This location suggests that corrosion products are not uniform and whatever has formed possibly dissolve in the solution, thus, a lower amount of O is found. However, once the EDS was taken at point 2, the amount of Na and Cl decreased dramatically. It means that the coagulated corrosion products (point 1) are mostly NaCl which are locally attacked to induce the corrosion reaction. However, K and Ca are decreased but the O content is increased. It is attributed to the formation of corrosion products/passive film. Moreover, the surface coverage area of point 2 is lower than 1 in Figure 8b, thus, the corrosion reaction is greater resulting in the decrease in *R_p_* and *R_o_*.

The EDS analysis of steel rebar sample exposed to 3% inhibitor-containing SCP + 3.5 wt% NaCl solution is carried out at two points as shown in Figure 8d. From point 1, it can be seen that the dendritic structure mostly contains O, Na, and Cl in greater amount. This result suggests that O is in significant amount attributed to the formation of some oxides of iron and phosphate. The presence of P reveals the formation of FePO_4_. The presence of N, i.e., 0.71% confirms that N is still present in the solution which comes from the NH_4_H_2_PO_4_ inhibitor. This result suggests that NH_4_OH (Equation (7)) is still present in SCP + 3.5 wt% NaCl solution even after 120 h of exposure. The Na and Cl amount is lower than the sample exposed to the bare solution. Thus, it can be said that Cl^−^ ions are not able to break the film. Na, Cl, Ca, and K are attributed to the composition of SCP solution. Moreover, Ca and K are in nominal amount. The EDS taken at point 2 (plain surface in Figure 8d) shows the greater amount of O, i.e., 6.15% while the sample exposed to bare solution shows 2.55%. The greater amount of O at point 2 in Figure 8d reveals the formation of iron oxide/hydroxide as well as the presence of P confirms the phosphate with Fe. There is very less amount of Na, Cl, K, and Ca owing to the composition of pore solution. The presence of O, P, and Fe reveals that 3% inhibitor may contain iron oxide/hydroxide and iron phosphate. Therefore, it is necessary to confirm the phases formed onto the steel rebar surface as corrosion products/passive film after 120 h of exposure in SCP + 3.5 wt% NaCl solution. In the subsequent paragraph, the nature of passive/oxide film characterized by Raman spectroscopy has been discussed.

#### 3.5.2. Raman Spectroscopy

**Figure 9:**
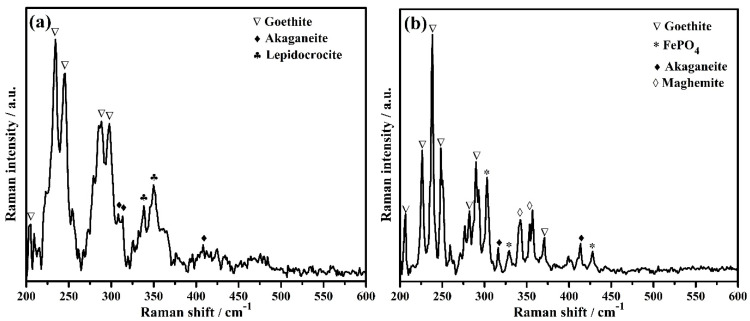
Raman spectra of steel rebar after 120 h exposure in (**a**) bare and (**b**) 3% inhibitor-added SCP + 3.5 wt% NaCl solutions.

The phases formed on steel rebar surface after 120 h of exposure in bare and 3% inhibitor-containing SCP + 3.5 wt% NaCl solutions are identified by Raman spectroscopy and the results are shown in Figure 9a,b, respectively. The steel rebar exposed to the bare solution formed goethite (α-FeOOH), akaganeite (β-FeOOH), and lepidocrocite (γ-FeOOH). The goethite [71,72] formed onto the steel rebar surface exposed to the bare solution is found to be at 205, 234, 245, 288, and 297 cm^−1^ (Figure 9a, Table 5), whereas, akaganeite [71] at 308, 314, and 410 cm^−1^ (Figure 9a, Table 5). The attribution of each phase is shown in Table 5 [71,72,73,74,75]. Akaganeite is mostly found in chloride environment and it is a chloride bearing iron oxide-hydroxide. Lepidocrocite [71,73] at 338 and 349 cm^−1^ (Figure 9a, Table 5) is a very unstable iron oxide-hydroxide of corrosion products and it is being formed during the initial corrosion process. It is believed that once lepidocrocite has formed, the corrosion reaction would be continued unless it transforms into stable phases. The presence of lepidocrocite as corrosion products onto the steel rebar surface exposed to the bare solution reveals the corrosion process to be continued even after 120 h of exposure. Once the 3% inhibitor is added in SCP + 3.5 wt% NaCl solution, two another phases, i.e., maghemite (γ-Fe_2_O_3_) and FePO_4_ (tertiary iron phosphate) are formed along with goethite and akaganeite onto the steel rebar surface. The presence of FePO_4_ as passive film corroborates with our assumption and mechanism as described in Equation (8). The goethite [71,72] is found at 206, 226, 238, 248, 282, 290, and 371 cm^−1^ (Figure 9b, Table 5). The number of peaks and intensity of goethite found on the steel rebar surface exposed to 3% inhibitor-added solution are greater compared to the bare solution. Along with goethite, maghemite [71] at 342 and 353 cm^−1^ (Figure 9b, Table 5) as well as FePO4 [74,75] at 303, 329, and 428 cm^−1^ (Figure 9b, Table 5) reveal the formation of very protective oxide/passive films which stifle the attack of Cl^−^ ions. Akaganeite [71] is found at 316 and 413 cm^−1^ (Figure 9b, Table 5). The presence of maghemite and FePO_4_ as oxide/passive film on steel rebar surface exposed to 3% inhibitor-containing SCP + 3.5 wt% NaCl solution lead to corrosion resistance. Thus, the highest *R_p_* and *R_o_* values are found after 120 h of exposure. Maghemite and FePO_4_ are sparingly soluble, thus, they provide protection to the steel rebar against corrosion in SCP + 3.5 wt% NaCl solution and maintain its efficiency around 83% (Table 3).

## 4. Conclusions

The addition of ammonium phosphate monobasic in SCP + 3.5 wt% NaCl solution improved the corrosion resistance performance of steel rebar at prolonged exposure periods. Once the 3 *v*/*v*% of 1 M ammonium phosphate monobasic inhibitor was added in SCP + 3.5 wt% NaCl solution, the *E_corr_* of steel rebars shifted towards a nobler direction and *i_corr_* decreased significantly. It shows 97.62% inhibition efficiency after 1 h of exposure. Moreover, the steel rebar exposed to 3% inhibitor-added SCP + 3.5 wt% NaCl solution exhibited ennobling in OCP as well as an increase in the *R_p_* and *R_o_* values with exposure periods. This sample maintained 82.82% efficiency after 120 h of exposure owing to the formation of protective passive film. The characterization of passive film by SEM confirmed the formation of uniform dendritic structure which were identified as maghemite and FePO_4_ by Raman spectroscopy.

## Figures and Tables

**Table 1:** Electrochemical parameters of steel rebars obtained from fitting of potentiodynamic plots in Tafel regions after 1 h of exposure in SCP + 3.5 wt% NaCl solution with and without inhibitor.

Sample ID	*E_corr_* (mV) vs. SCE	*i_corr_* (µA·cm^−2^)	Corrosion Rate (µm·year^−1^)	Efficiency (%)
Bare	−486 (±5)	2.52 (±0.20)	29.28 (±1.46)	0
1%	−439 (±2)	0.33 (±0.01)	3.83 (±0.33)	86.90 (±6.52)
2%	−401 (±2)	0.18 (±0.03)	2.09 (±0.06)	92.86 (±4.18)
3%	−379 (±1)	0.06 (±0.002)	0.70 (±0.02)	97.62 (±5.97)

**Table 2:** Adsorption parameters of inhibitor.

Surface Coverage (θ)			Isotherm Method	*K_ads_* (M^−1^)	∆*G*^0^*_ads_* (kJ mol^−1^)
1% Inhibitor	2% Inhibitor	3% Inhibitor			
0.869	0.9286	0.9762	Langmuir	500	−24.92
			Freundlich	1.40	−10.61

**Table 3:** Electrochemical parameters obtained by EIS fitting of steel rebars exposed to bare and inhibitor-added SCP + 3.5 wt% NaCl solutions for different durations.

Time (h)	Sample ID	Electrochemical Parameters							Efficiency (%)
		*R_s_* (Ω·cm^2^)	*R_p_* (kΩ·cm^2^)	*CPE1*		*R_o_* (kΩ·cm^2^)	*CPE2*		
				*Q*_1_ (1 × 10^−5^) (Ω^−1^·cm^−2^·s^n^)	*n*_1_		*Q*_2_ (1 × 10^−5^) (Ω^−1^·cm^−2^·s^n^)	*n*_2_	
**1**	**Bare**	9.96 (±0.40)	1.96 (±0.15)	21.0 (±1.89)	0.78 (±0.06)	0.87 (±0.07)	232.8 (±20.95)	0.70 (±0.04)	0
	**1% inhibitor**	10.55 (±0.84)	8.04 (±0.65)	10.1 (±0.56)	0.82 (±0.03)	2.61 (±0.10)	34.1 (±2.05)	0.74 (±0.03)	66.67 (±4.00)
	**2% inhibitor**	10.72 (±0.70)	10.87 (±0.67)	8.6 (±0.48)	0.87 (±0.06)	9.10 (±0.82)	12.8 (±0.38)	0.80 (±0.05)	90.44 (±6.91)
	**3% inhibitor**	11.75 (±0.82)	10.41 (±0.86)	8.8 (±1.58)	0.86 (±0.07)	8.21 (±0.29)	14.89 (±0.91)	0.78 (±0.02)	89.40 (±3.58)
**24**	**Bare**	11.48 (±0.52)	3.29 (±0.22)	16.6 (±1.35)	0.80 (±0.05)	2.16 (±0.09)	182.4 (±11.67)	0.73 (±0.03)	0
	**1% inhibitor**	18.21 (±0.75)	3.87 (±0.08)	15.5 (±1.24)	0.81 (±0.02)	2.53 (±0.05)	95.0 (±2.00)	0.74 (±0.04)	14.62 (±1.09)
	**2% inhibitor**	13.62 (±0.66)	6.63 (±0.23)	12.3 (±1.16)	0.85 (±0.02)	4.23 (±0.33)	21.5 (±0.43)	0.75 (±0.04)	48.94 (±2.20)
	**3% inhibitor**	12.87 (±0.71)	11.15 (±0.66)	7.3 (±0.31)	0.87 (±0.03)	14.20 (±0.47)	6.7 (±0.19)	0.88 (±0.03)	84.79 (±3.82)
**120**	**Bare**	11.12 (±0.39)	1.88 (±0.04)	23.8 (±2.09)	0.76 (±0.02)	2.66 (±0.14)	155.7 (±12.76)	0.73 (±0.04)	0
	**1% inhibitor**	9.39 (±0.33)	1.44 (±0.03)	30.6 (±2.35)	0.75 (±0.02)	2.37 (±0.15)	165.2 (±4.63)	0.71 (±0.04)	−13.92 (±0.46)
	**2% inhibitor**	10.30 (±0.07)	6.43 (±0.16)	12.9 (±0.20)	0.85 (±0.01)	4.21 (±0.14)	22.3 (±2.03)	0.75 (±0.06)	36.82 (±2.03)
	**3% inhibitor**	11.86 (±0.65)	12.51 (±0.75)	5.9 (±0.24)	0.88 (±0.03)	15.48 (±0.56)	4.7 (±0.13)	0.89 (±0.03)	82.82 (±5.13)

**Table 4:** EDS of steel rebar sample after 120 h of exposure in bare and 3% inhibitor-containing SCP + 3.5 wt% NaCl solution.

Sample ID	Point	O	Na	Cl	K	Ca	N	P	Fe
Bare	1	1.40	50.10	42.76	2.34	1.49	0	0	Balance
	2	2.55	2.16	1.77	0.35	0.19	0	0	Balance
3%	1	16.91	34.81	30.63	0.61	0.08	0.71	1.21	Balance
	2	6.15	0.72	0.33	0.27	0.14	1.12	0.35	Balance

**Table 5:** Attribution of Raman peaks formed on steel rebar surface after 120 h exposure in bare and 3% inhibitor-added SCP + 3.5 wt% NaCl solutions.

Sample ID	Raman Shift (cm^−1^)	Attribution	Reference
Bare	205, 234, 245, 288, 297	Goethite (α-FeOOH)	[71,72]
	308, 314, 410	Akaganeite (β-FeOOH)	[71]
	338, 349	Lepidocrocite (γ-FeOOH)	[71,73]
3% inhibitor	206, 226, 238, 248, 282, 290, 371	Goethite (α-FeOOH)	[71,72]
	316, 413	Akaganeite (β-FeOOH)	[71]
	342, 353	Maghemite (γ-Fe_2_O_3_)	[71]
	303, 329, 428	FePO_4_ (tertiary iron phosphate)	[74,75]
